# Benefits of sustained physical activity from middle age to early old age on quality of life in early old age

**DOI:** 10.1038/s41598-022-20431-0

**Published:** 2022-09-30

**Authors:** Mikyung Lee, Hyeonkyeong Lee, Kijun Song, Young-Me Lee

**Affiliations:** 1 College of Nursing, Brain Korea 21 FOUR Project, Yonsei University, Seoul, Republic of Korea; 2 College of Nursing and Mo-Im Kim Nursing Research Institute, Yonsei University, Seoul, Republic of Korea; 3 School of Nursing, DePaul University, Chicago, IL USA

**Keywords:** Health care, Risk factors

## Abstract

This study aimed to examine changes in physical activity (PA) over time (2009–2017) in the same participants and to determine an association between changes in PA and health-related quality of life (HRQoL) in early older adults (*n* = 994) using data from the Korea Health Panel Survey. HRQoL was measured using the EuroQol system, and the amount of PA was grouped into four activity levels: remained inactive, became inactive, became active, and remained active. The association of changes in PA over 8 years with HRQoL was examined using logistic regression analysis while controlling for socioeconomic and behavioral factors. Total PA decreased from 1859.72 ± 1760.01 MET-minutes in 2009 to 1264.80 ± 1251.14 MET-minutes in 2017 (*P* < 0.001). In 2017, 142 (14.3%) remained inactive, whereas 419 (42.2%) remained active. Participants who remained inactive in early old age were more likely to be in the lowest 10% HRQoL of the sample (odds ratio = 1.95, 95% confidence interval = 1.09–3.48). These findings indicate that health education and promotion must be prioritized for middle-aged adults, who are relatively inactive, so that they increase their current PA and improve their HRQoL to maximize the benefits of PA in old age.

## Introduction

Older adults are the fastest-growing segment of the South Korean population, making South Korea the second-fastest aging country after Japan since 2000. This rapid increase in the number of older adults is a result of a dramatic decline in fertility rates and an increase in life expectancy in South Korea^1^. The older population is expected to more than double over the next 20 years, from 8.03 million in 2020 to 16.66 million in 2040^2^. The healthy life expectancy in Korea is 73.1 years, which has a 10.2-year gap relative to Korea’s life expectancy of 83.3 years, and the gap is wider than the average gap of 8.9 years in the Western Pacific region to which Korea belongs^3,4^. With the rapid growth of the older population, interest in healthy aging and quality of life (QoL) in old age has also increased, and the improvement of healthy life expectancy along with the increase in life expectancy has emerged as an important national policy goal^5,6^.

In particular, quality of life in older adults is often negatively affected due to increased frailty^7^. Meta-analyses have shown that there is a consistent inverse correlation between frailty, prefrailty, and QoL in community-dwelling older people, and these studies have argued that interventions to reduce frailty are needed to improve QoL^8^. Physical activity (PA) is widely known as a preventive intervention for frailty^9^. PA also has an important role in the well-being of aging people^10^. Studies have reported that PA in early old age is associated with positive health outcomes, such as improvement of metabolic syndrome, reduction of depression, improvement of cognitive function, and prevention of dementia^11,12^. According to a systematic literature review, physically active older adults demonstrated higher levels of health-related quality of life (HRQoL)^13^. Additionally, the two studies on older adults in Korea also showed consistent results: those who performed moderate- or high-intensity PA had increased QoL compared with those who were not physically active^14,15^. The PA of early old-age (between 55 and 64 years) workers was found to have a significant impact on improving HRQoL^16^.

Despite the benefits of regular PA in older adults, the prevalence of the minimum recommended exercise level (150 min per week of moderate-intensity PA or 75 min per week of vigorous-intensity PA, as suggested by the World Health Organization^17^) was only 37.6% among older Korean adults in 2020, according to the Living the Profiles of Older People Survey^18^. The study reported another finding with regard to Korean older adults: the prevalence of older adults engaged in physical activity for 10 min or more over a week decreased to 53.7% in 2020 compared to 68.0% in 2017^18^. Furthermore, as a result of a telephone survey of changes in the frequency or time of indoor and outdoor physical activity for the older Korean adults (65 years and over) in 2020 after COVID-19, it was reported that the total amount of exercise decreased compared to before COVID-19^19^.

The problem of low PA in older adults should be addressed by encouraging a lifestyle habit starting in middle age. Physically active middle-aged population under 65 years of age had a 22% reduction in mortality over the follow-up period (a median of 12.5 years) than those who were physically inactive^20^. In another study conducted in Australia, adults who aged 45–64 years engaging in some vigorous activity (30% or more of total moderate to vigorous physical activity as vigorous activity) approximately 22–25% more likely to have decreased mortality after 6–8 years^21^, compared with the group that was not engaging in vigorous physical activity. A cohort study followed women ages 35–60 and men ages 45–60 for 3 and 10 years and analyzed their QoL in early old age^22,23^. This study reported that an increase in physical activity for several years was highly correlated with improvement in QoL. Another longitudinal study also reported that middle-aged (40–59 years) adults who had high levels of PA continued to maintain high levels of PA even in old age^24^, which further emphasizes the importance of PA adherence before old age. As aforementioned, there is evidence that a higher level of PA in middle age is associated with a higher HRQoL in old age; however, an understanding of the effect on HRQoL in old age according to the level of PA in middle age is still lacking. Thus, the objectives of this secondary data analysis study are to examine the changes in PA over time, from middle age to early old age, in the same participants and to determine the association of changes in PA with HRQoL among the early older adults.

## Methods

### Research design

Secondary data analysis was conducted to examine the changes in PA over time associated with participants’ recorded quality of life as middle-aged and older adults. This design was appropriate for assessing changes in PA measured at two points in time, 2009 and 2017, in the same participants and for observing how the level of PA changed from middle age to old age and the association of this change with quality of life.

### Sample and data

The Korea Institute for Health and Social Affairs and the National Health Insurance Service have formed a consortium to jointly conduct the nationwide Korea Health Panel Survey (KHPS) V.1.6 to obtain basic data regarding healthcare utilization, health expenditure level, and health-related behaviors^25^. The KHPS is a longitudinal study that provides nationally representative data. Using probability-proportional stratified two-stage cluster sampling, the registration census was used as an extraction frame, and general households in 17 cities and provinces were targeted. After systematic sampling by region, household members belonging to each household were asked to complete the survey questionnaire. The survey has been conducted annually since 2008, but the database from 2008 was excluded because it does not contain data for individual health-related behavioral factors such as PA, which is a key variable in our study. In 2009, additional parameters, including smoking, drinking, PA, and quality of life, were added to the survey. KHPS data do not include any personal identifiers and the survey complies with the Personal Information Protection Act and the Statistics Act. Participants were fully informed that their data are public and can be used for research purposes. The authors obtained “Consent to Use Data” according to the KHPS guidelines and regulations. The use of data from the KHPS was approved by the Institutional Review Board of Yonsei University Health System (no. Y-2020-0232).

Initial review of the retrospective data collected from the KHPS revealed that 7866 households participated in the panel survey in 2008. In 2009, when questions related to health lifestyle were added, 6798 households participated. Tracking the IDs of participants in the 2017 survey revealed that 1322 persons ages 60–67 years had also participated in 2009. Those who participated in both surveys, in 2009 (ages 52–59 years, i.e., middle-aged adults) and 2017 (ages 60–67 years, i.e., early older adults), were selected to determine any changes in PA over time and the impact of those changes on quality of life as reported by these middle-aged to older adults. Participants who responded to the survey by indicating disability or sickness and who answered “yes” to the question of whether they had needed to “lie down almost all day due to illness or injury” in the last month were excluded. This brought the total to 1090 participants. After excluding those with missing data on quality of life in 2009 (*n* = 68) and 2017 (*n* = 28), data from 994 participants were included in the analysis.

### Measures and variables

#### Physical activity

PA was measured using the International Physical Activity Questionnaire (IPAQ), a structured questionnaire tool that calculates the sum of the amount of PA using the average number of days and duration per day of high- and moderate-intensity activity as well as walking activity for at least 10 min during the previous week^26^. The amount of PA was calculated using the metabolic equivalent of task (MET, in minutes) score for high-intensity activity (MET ≥ 8.0), moderate-intensity activity (MET of 4.0), and walking (MET of 3.3). In the KHPS, the duration of PA is a categorical variable, according to convention in previous studies^27^; thus, it was converted to the median value of the range of each response category. For example, “more than 20 min to less than 30 min” was converted to 25 min.

The amount of PA was classified into three groups (low, moderate, and high) according to the classification criteria suggested by the IPAQ^28^. The high level indicated either high-intensity activity 3 days/week that accumulated at least 1500 MET-minutes/week or 5 days of any combination of walking, moderate-intensity, and high-intensity activities achieving at least 3000 MET-minutes/week. The moderate level indicated 30 min of moderate-intensity activity 5 days/week, 20 min of vigorous activity 3 days/week, or a combination achieving at least 600 MET-minutes/week^26^. The low level indicated meeting neither moderate nor high PA criteria^26^.

In this study, the change in PA level between 2009 and 2017 was classified into four phases using the three PA groups used by Hamer et al.^28^. The three groups (low, moderate, and high) were changed to the binary variables inactive (low) and active (moderate or high). Then, the change in PA level during the 8-year follow-up period was divided into four levels: remained inactive, became inactive, became active, and remained active.

#### Health-related quality of life

HRQoL was measured using the EuroQol quality-of-life system, 5-dimension, 3-level version (EQ-5D-3L). The EQ-5D was developed by the EuroQol Group, which was established in 1987^29^. It assesses five dimensions of health: mobility, self-care, usual activity, pain/discomfort, and anxiety/depression. Each dimension is measured on a nominal scale and consists of three questions representing three levels of functioning: no problem (1), some problem (2), and serious problem (3). The three questions in the five health dimensions were derived from 243 combinations of responses to each question regarding different health levels^24^. A EQ-5D index can be calculated using a formula that provides weights to each of the dimensions. In this study, the EQ-5D index was calculated using the Korean quality-weighting formula, and a value closer to 1 indicates a better HRQoL^30^. In this study, because there was no fixed cutoff point to define poor quality of life, a cutoff point from another study was used^31^: the lower 10% fractile was used to indicate poor HRQoL.

#### Personal factors

Personal factors—including gender, marital status, educational level, economic activity, presence of chronic diseases, body mass index (BMI), smoking status, and alcohol consumption in 2009—were measured and considered as covariates. We assumed that personal factors in 2009 would affect the HRQoL after 8 years and confirmed the results of this assumption. We did not include personal factors in 2017. BMI (weight [kg]/height [m]^2^) was divided into three sequence scales: underweight (< 18.5), normal weight (18.5–24.9), and overweight (≥ 25). Smoking status was identified as “no,” “past,” or “current.” Alcohol consumption was classified as “no,” “less than three times a month,” “less than three times a week,” or “almost every day.”

### Statistical analyses

All data and statistical analyses were performed with SPSS version 25.0 (IBM Corp., Armonk, NY, USA) for Windows and Stata IC version 16 (Stata Corp, College Station, TX, USA). The descriptive statistics were calculated to analyze the demographics and key variables in 2009 related to HRQoL in 2017. To compare the level of PA and HRQoL in middle (2009) and early old age (2017), including health-related behavioral factors and demographic variables, the χ^2^ test and *t*-test were performed. Lastly, logistic regression analysis was used to identify factors independently associated with the lower 10% quality of life in 2017, including BMI, chronic diseases, demographic characteristics, and changes in PA. We analyzed odds ratios (ORs) and 95% confidence intervals (CIs). Results with a *P*-value ≤ 0.05 were deemed statistically significant. The effect size between PA change (four levels: remained inactive, became inactive, became active, and remained active) and HRQoL was calculated using the test of Cramer’s V coefficient (CVC) with a chi-square for goodness of fit and contingency^32^.

## Results

Comparison of the HRQoL in a dichotomous manner based on the lower 10% (Table 1) showed that the proportion of HRQoL in the lower 10% was 13.4% for women (*n* = 78), which was 6.4% higher than for men (*n* = 29) (χ^2^ = 10.17, *P* < 0.001). Regarding marital status, the percentage of HRQoL in the lower 10% was the highest at 21.3% (*n* = 10) in divorced/separated participants and was statistically significant (χ^2^ = 8.38, *P* = 0.039). In terms of level of education, the percentage of HRQoL in the lower 10% accounted for 14.4% (*n* = 35) of middle school graduates and was statistically significant (χ^2^ = 15.21, *P* = 0.002). Compared with participants with no chronic disease (6.6%; *n* = 18), the proportion of HRQoL in the lower 10% increased to 12.3% (*n* = 89) and was statistically significant (χ^2^ = 6.59, *P* = 0.010). In underweight participants, the percentage of HRQoL at the lower 10% was the highest at 33.3% (*n* = 5) and was statistically significant (χ^2^ = 11.70, *P* = 0.003). Economic activity, smoking, and drinking (consumption of alcohol) were not statistically significant. Regarding the change of level of PA, the percentage in the lower 10% of HRQoL was the highest at 16.9% (*n* = 24) in the “remained inactive” participants and was statistically significant (χ^2^ = 15.21, *P* = 0.002). The effect size between change of level of PA and the lower 10% of HRQoL, calculated by Cramer’s V coefficient, was 0.39.

**Table 1:** Comparison of the lower 10% of HRQoL (2017) according to general characteristics (2009).

Variables	Lower 10% of HRQoL		χ^2^	*P*-value
	Yes	No		
**Gender**			10.17	< 0.001
Men	29 (7.0)	383 (93.0)		
Women	78 (13.4)	504 (86.6)		
**Marital status**			8.38	0.039
Married	80 (9.6)	751 (90.4)		
Single	1 (16.7)	5 (83.3)		
Widow/widower	16 (14.5)	94 (85.5)		
Divorce/separated	10 (21.3)	37 (78.7)		
**Education**			15.21	0.002
≤ Elementary school	42 (13.9)	260 (86.1)		
Middle school	35 (14.4)	208 (85.6)		
High school	24 (7.6)	291 (92.4)		
≥ University	6 (4.5)	128 (95.5)		
**Economic activity**			3.39	0.066
Yes	69 (9.6)	647 (90.4)		
No	38 (13.7)	240 (86.3)		
**Chronic diseases**			6.59	0.010
Yes	89 (12.3)	634 (87.7)		
No	18 (6.6)	253 (93.4)		
**BMI**			11.70	0.003
< 18.5	5 (33.3)	10 (66.7)		
18.5–24.9	61 (9.1)	607 (90.9)		
≥ 25.0	41 (13.2)	270 (86.8)		
**Smoking**			3.43	0.188
No	74 (11.7)	557 (88.3)	631	
Past	12 (6.9)	162 (93.1)	174	
Current	21 (11.1)	168 (88.9)	189	
**Drinking**			2.86	0.414
No	32 (10.8)	265 (89.2)		
1–3 times/month	52 (12.3)	371 (87.7)		
1–3 times/week	15 (7.8)	177 (92.2)		
Almost every day	8 (9.8)	74 (90.2)		
**Change of level of physical activity**			15.21	0.002
Remained inactive	24 (16.9)	118 (83.1)		
Became inactive	39 (14.2)	236 (85.8)		
Became active	10 (6.3)	148 (93.7)		
Remained active	34 (8.1)	385 (91.9)		
Total	107 (10.8)	887 (89.2)		

Values are presented as *n* (%).

*HRQoL* health-related quality of life, *BMI* body mass index.

**Figure 1:**
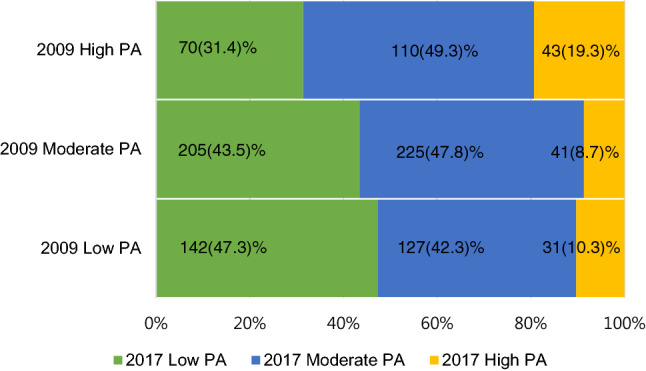
Physical activity (PA) categories in 2009 and 2017.

Dividing PA in 2009 and 2017 into low, moderate, and high based on the IPAQ scores (Fig. 1) revealed that 142 (47.3%) participants who had low PA in 2009 maintained low PA in 2017. Meanwhile, 225 (47.8%) participants who had moderate PA in 2009 maintained moderate PA in 2017, and 110 (49.3%) participants who had high PA in 2009 reduced to moderate PA in 2017.

The changes in PA and HRQoL in 2009 and 2017 are presented in Table 2. Vigorous-intensity activity had sharply declined from 630.50 ± 1142.08 MET-minutes in 2009 to 267.81 ± 742.80 MET-minutes in 2017. Walking had slightly decreased from 745.09 ± 574.41 MET-minutes in 2009 to 685.36 ± 565.77 MET-minutes in 2017. The total PA also sharply declined from 1859.72 ± 1760.01 MET-minutes in 2009 to 1264.80 ± 1251.14 MET-minutes in 2017. The HRQoL decreased from 0.96 ± 0.07 in 2009 to 0.94 ± 0.08 in 2017, and the decrease was statistically significant.

**Table 2:** Comparison of PA and HRQoL between 2009 and 2017.

Variables	2009	2017	*P*-value
**Amount of PA types**			
Vigorous intensity (MET-minutes)	630.50 ± 1142.08	267.81 ± 742.80	< 0.001
Moderate intensity (MET-minutes)	484.12 ± 657.26	311.63 ± 551.39	< 0.001
Walking (MET-minutes)	745.09 ± 574.41	685.36 ± 565.77	< 0.001
Total PA (MET-minutes)	1859.72 ± 1760.01	1264.80 ± 1251.14	< 0.001
HRQoL	0.96 ± 0.07	0.94 ± 0.08	< 0.001

Values are presented as mean ± standard deviation.

*PA* physical activity, *HRQoL* health-related quality of life.

Table 3 presents the results of the binary logistic regression analysis based on the lower 10% in HRQoL. Analysis of the changes in the PA categories in 2009 and 2017 showed that the probability of HRQoL belonging to the lower 10% increased when the participants remained inactive (OR = 1.95, 95% CI = 1.09–3.48) or became inactive (OR = 1.67, 95% CI = 1.01–2.75). Among the sociodemographic characteristics in 2009, those who were divorced/separated (OR = 2.39, 95% CI = 1.12–5.12), were middle school graduates (OR = 2.64, 95% CI = 1.04–6.69), or had chronic diseases (OR = 1.83, 95% CI = 1.05–3.18) were more likely to belong to the lower 10% in 2017.

**Table 3:** Effect of changes in the PA groups from 2009 to 2017 and general characteristics in 2009 on lower 10% of HRQoL in 2017.

Variables	Odds ratio	95% CI	*P*-value
**Change of level of physical activity**			
Remained inactive	**1.95**	1.09–3.48	0.025
Became inactive	**1.67**	1.01–2.75	0.045
Became active	0.66	0.32–1.40	0.280
Remained active	Ref.		
**Gender**			
Men	Ref.		
Women	1.55	0.95–2.53	0.076
**Marital status**			
Married	Ref.		
Single	4.07	0.40–41.54	0.240
Widow/widower	1.18	0.64–2.18	0.589
Divorce/separated	**2.39**	1.12–5.12	0.025
**Education**			
≤ Elementary school	2.40	0.95–6.02	0.063
Middle school	**2.64**	1.04–6.69	0.041
High school	1.46	0.57–3.76	0.436
≥ University	Ref.		
**Chronic diseases**			
Yes	**1.83**	1.05–3.18	0.033
No	Ref.		
**BMI**			
< 18.5	2.60	0.78–8.63	0.119
18.5–24.9	0.72	0.47–1.12	0.147
≥ 25.0	Ref.		

*PA* physical activity, *95% CI* 95% confidence interval, *BMI* body mass index.

Boldface P-values indicate significance level < 0.05.

## Discussion

The current study examined the longitudinal changes in PA that influenced HRQoL in middle age and early old age over 8 years of follow-up. The results showed that older adult participants who continued to be inactive or became inactive, compared with their PA during middle age, had an increased likelihood of worsened quality of life.

PA improves the HRQoL by improving physical function and lowering the risk of chronic diseases such as obesity, thereby inducing fundamental health benefits^33^, as reported in earlier studies. An analysis of older adults 65 years and older in Korea showed that the physically active group had lower pain, pain interference, and fatigue than the inactive group^34^. HRQoL decreased linearly with decreasing PA, with participants showing weakened physical and mental health, including bodily pain, physical role limitation, and emotional role limitation^35^. Consistent with the findings of enhanced HRQoL with PA^10,17^, the current study observed a positive effect of PA on the quality of life in old age while re-emphasizing the importance of being physically active throughout life.

The difference and strength of this study from previous studies is that it considered the changes in the PA level of the older adults during the 8-year follow-up. In a similar study^20^ with a large sample of middle-aged and older Australian adults, the group that reported any PA or vigorous activity had associated risk reductions in mortality as well as the positive health effects of PA with a mean follow-up of 6.5 years. In a large study in England^28^, both becoming and remaining active were associated with healthy aging, compared with remaining inactive, over an 8-year follow-up, and the impact was higher in the group that remained active than in the group that became active. This suggests that the attention of practitioners is required to achieve the goal of extending healthy life rather than simply extending life expectancy in an aging society. The recommended amount of PA for the adult population should be more actively approached from the viewpoint of not only preventing chronic diseases but also preparing for a healthy life in old age.

The second difference and strength of this study compared to previous studies is that we selected early older adult participants and measured their PA level at two stages of life: one measurement at middle age (ages 52–59) and one at early old age (ages 60–67). Our study focused on PA measured over time from a cohort in their 50s and 60s, whereas previous studies followed only one specific age group, such as people in their 60s^10^, or focused on people 65 years of age or older^14,36^. Some other studies only focused on people in their 70s and older, who usually reported a lower level of QoL^38^. However, our study was able to track changes in PA from middle age to early old age, providing a better understanding of how QoL in early old age is affected by middle age.

The phenomenon of the rapidly increasing aging population led health researchers to give more attention to frailty, which is one of the determinants of HRQoL. The high prevalence of frailty, a dynamic state of experiencing a decline in at least one of the human function domains (physical, psychological, and social)^7^, is among one of the major problems of older adults, and earlier studies showed the negative influence of frailty on the HRQoL of older adults^37,38^. Importantly, PA is considered an important component that can help prevent or improve frailty in older adults, as evidenced by 19 interventional studies^9^. In this study, the effect of changes in PA with frailty as an outcome variable was not analyzed, but in light of the relationship with HRQoL, the changes in PA from middle age to old age may have a relationship with frailty. Hence, we suggest that future research examine the state of frailty according to the amount of PA.

The effect size was calculated for the correlation between PA and HRQoL in the present study because even if the statistical methods are different between studies, they can be converted into a common unit and compared. The effect size was 0.39 in this study, which is comparable to the effect size of 0.26 found in a meta-analysis on physical activity and quality of life in older adults^39^. Thus, our study provides further support for the positive effect of engaging in physical activity on HRQoL in older adults.

Despite the benefits of PA, most people find it difficult to start and maintain PA^40,41^. Health behavior is not easy to modify because of long-term life habits; furthermore, it is difficult to influence simply by value judgment and norms that say PA is beneficial to health^41^. As a result, a previous study stated that a step-by-step intervention program is important to improve motivation, intention, and practice^42^. A 12-week intervention study with older Korean Americans^42^ showed that PA, walking endurance, and flexibility were higher in the group that received a PA motivational intervention that incorporated social support, empowering education, and motivational education. A systematic literature review of motivational studies for PA using behavior change techniques (BCTs) showed the effectiveness of BCT in improving PA^43^. Therefore, we recommend a motivational method through BCT for a PA intervention for older adults.

Similar to earlier studies, the current study revealed the influence of socioeconomic status on HRQoL among older adults. Recent literature showed that a lower level of education was strongly related to a lower reported level of physical functioning^44^ and daily life activities^45^, and a decrease in quality of life^44–46^. According to the Korea National Health and Nutrition Examination Survey published recently^47^, older adults with a higher level of education showed higher adherence to walking “more than 5 days a week and more than 30 min a day,” whereas older adults with a low level of education (did not graduate from elementary school) did not practice recommended walking. This suggests that interventions aimed at changing individuals’ behavior to increase physical activity should be prioritized for lower socioeconomic status groups to reduce existing health disparities.

There are some limitations to the study that deserve mentioning. Because secondary data were obtained from people at home, excluding members from branch or dead households at the time of the survey, persons with low quality of life and high probability of dying from severe illness may have been excluded. As such, reporting bias may have occurred. Furthermore, we did not include the changes in individual characteristics that would affect participants’ HRQoL after 8 years rather we focused on the cumulative effect of PA on HRQoL. Therefore, the changes of various individual characteristics, health behaviors, and circumstances need to be considered as factors affecting HRQoL in future research.

## Conclusion

This study showed that most of the participant’s HRQoL scores were ≥ 0.9 (range was 0 [lowest] to 1 [highest]). We recommend comparing the amount of PA according to the five dimensions of the EuroQol system or analyzing the quality of life more discriminatively through other research tools. In addition to analyzing the effect of PA frequency and intensity on quality of life in more detail, future studies should analyze both aerobic PA and strength exercise because both have a positive effect on health in older adults. Explanatory variables for the association between PA and quality of life through mediators such as self-efficacy and social support should be identified. In the survey data of this study, the responses regarding PA were subjective and memory-dependent; thus, recall bias may have occurred. Therefore, we recommend that more objective data be collected through wearable devices and that experimental research be conducted to supplement the evidence for PA and quality of life.

## Data Availability

The data that support the findings of this study are available from KHPS v.1.6. Restrictions apply to the availability of these data, which were used under license for the current study and so are not publicly available. Data are, however, available from the authors upon reasonable request and with permission from the Korea Institute for Health and Social Affairs.
